# Magnetic resonance spectroscopic imaging in gliomas: clinical diagnosis and radiotherapy planning

**DOI:** 10.1259/bjro.20190026

**Published:** 2020-04-06

**Authors:** Maria Elena Laino, Robert Young, Kathryn Beal, Sofia Haque, Yousef Mazaheri, Giuseppe Corrias, Almir GV Bitencourt, Sasan Karimi, Sunitha B Thakur

**Affiliations:** 1 Department of Radiology, Memorial Sloan Kettering Cancer Center, 1275 York Ave, New York, NY 10065, USA; 2 Department of Radiology, Humanitas Research Hospital, Via Alessandro Manzoni 56, 20089, Rozzano, MI, Italy; 3 3 Department of Radiation Oncology, Memorial Sloan Kettering Cancer Center, 1275 York Ave, New York, NY 10065, USA; 4 4 Department of Medical Physics, Memorial Sloan Kettering Cancer Center, 300E 66th Street, New York, NY 10065, USA; 5 Department of Radiology, University of Cagliari, 40 Via Università, 09124 Cagliari, Italy; 6 Department of Imaging, A.C.Camargo Cancer Center, São Paulo SP, Brazil

## Abstract

The reprogramming of cellular metabolism is a hallmark of cancer diagnosis and prognosis. Proton magnetic resonance spectroscopic imaging (MRSI) is a non-invasive diagnostic technique for investigating brain metabolism to establish cancer diagnosis and *IDH* gene mutation diagnosis as well as facilitate pre-operative planning and treatment response monitoring. By allowing tissue metabolism to be quantified, MRSI provides added value to conventional MRI. MRSI can generate metabolite maps from a single volume or multiple volume elements within the whole brain. Metabolites such as NAA, Cho and Cr, as well as their ratios Cho:NAA ratio and Cho:Cr ratio, have been used to provide tumor diagnosis and aid in radiation therapy planning as well as treatment assessment. In addition to these common metabolites, 2-hydroxygluterate (2HG) has also been quantified using MRSI following the recent discovery of *IDH* mutations in gliomas. This has opened up targeted drug development to inhibit the mutant *IDH* pathway. This review provides guidance on MRSI in brain gliomas, including its acquisition, analysis methods, and evolving clinical applications.

## Introduction

Primary malignant tumors of the brain represent 2% of all cancers in adults,^1^ with an annual global age-standardized incidence of approximately 3.7 cancers per 100,000 males and 2.6 cancers per 100,000 females.^2,3^ Recently, an updated classification model was proposed by the World Health Organization (WHO) for brain tumors to include not only histology but also isocitrate dehydrogenase (IDH) status and related genetic parameters where they are relevant.^4^ According to WHO, gliomas are categorized into four grades, determined by pathologic types of the tumor based upon histopathological characteristics such as cytological atypia, anaplasia, mitotic activity, microvascular proliferation, and necrosis.

Low-grade gliomas exhibit benign tendencies and portend a better prognosis for the patient but at the same time they have a uniform rate of recurrence and increase in grade over time.^5^ High-grade gliomas tend to grow rapidly and spread faster than tumors of a lower grade, and they carry a worse prognosis. Among low-grade lesions, the majority of the lesions are IDH mutant (oligodendroglioma, astrocytoma) and the rest are astrocytomas with IDH wild-type. Glioblastoma multiforme (GBM), a WHO Grade IV astrocytoma with key features of primary and secondary tumors, represents the most common malignant primary brain tumor in adults,^6^ with an incidence of nearly 15,000 cases each year in the United States.^7^ Among GBM lesions, 90% have no missense of IDH mutations. IDH mutant tumors have better prognosis compared with wild-type IDH have poor prognosis.^8^ The gold-standard treatment for GBM consists of surgery followed by radiation therapy (RT) and then concurrent and adjuvant temozolomide chemotherapy.^7^ RT is the most effective non-surgical form of cancer treatment.^9^ Precise delineation of both the RT target and areas of likely anatomic spread is particularly important to optimize local control of the disease and reduce the risk of neurological deficits from RT-related damage to the adjacent normal tissues.^10^ Brain cancer is a complex and heterogeneous disease, therefore posing varied clinical challenges in regard to treatment response assessment and prognostication. In this context, it is necessary to acquire a deeper understanding of the tumor properties that can affect accurate tumor delineation.

RT target volumes are determined based on MRI. Contrast-enhanced *T*_1_ weighted (CE-*T*_1_W) sequences are helpful to define the resection cavity^11^ whereas the *T*_2_ weighted fluid-attenuated inversion recovery (FLAIR) sequence is helpful to indicate areas of brain alteration from edema or tumor infiltration. However, conventional MRI is limited in certain aspects. While CE-*T*_1_W sequences are helpful for indicating areas with a compromised blood–brain barrier due to the presence of the tumor, they are limited for defining the extent of the disease as infiltrating tumor cells can be present centimeters away from the contrast-enhancing mass.^7,11^ Indeed, GBM recurs within 2 cm of the original CE-*T*_1_W tumor margins after therapy in nearly 80% of patients.^7^ Hence, by simply considering the area of contrast enhancement on CE-*T*_1_W as the macroscopic tumor and the surrounding hyperintensity on *T*_2_W/FLAIR images as the related edema, MRI represents an oversimplified approach that does not reflect the biologic and histologic characteristics of the lesion, and in the case of treated lesions, does not distinguish viable tumor from treatment-related changes.^12^

Compared with conventional MRI, advanced imaging techniques such as proton magnetic resonance spectroscopy imaging (MRSI) allow better characterization of the lesion with more accurate target definition in the pre-treatment setting. In the post-treatment setting, advanced imaging techniques also play an important role in treatment assessment as they facilitate the identification of tumor recurrence and its differentiation from treatment-related changes.^12^ Malignant brain tumors are characterized by rapid cellular proliferation through increased glycolytic metabolism, protein synthesis, and membrane synthesis.^12,13^ In this context, MRSI has been shown to provide information on tumor metabolism and heterogeneity, helping to differentiate regions of active and infiltrating tumor from normal brain tissue, vasogenic edema,^14^ or treatment-related changes.^15^

The aim of this review is to provide readers with the overview of MRSI and metabolite quantification in gliomas for cancer diagnosis, pre-operative radiotherapy planning, and treatment assessment.

## Magnetic resonance spectroscopy imaging

Conventional MRI detects and localizes the proton signals from fat and water molecules to produce images. Proton MRSI measures the chemical spectrum of the tissue, where individual resonance peaks represent metabolite concentrations from a specific region. Hence, MRSI can complement the anatomical information from conventional MRI to allow better tissue characterization.^16,17^ In MRSI, all observable metabolites can be characterized by their unique set of chemical shifts. Specifically, the chemical structure of the metabolite contributes to the position and characteristics of the metabolite peak on the MRS spectra, while the concentration of the metabolite contributes to the area under the peak. The metabolites measured, and the techniques typically used to measure them tend to be disease-specific. The most important and common MRS metabolites include N-acetyl aspartate (NAA), total choline compounds (Cho), total Creatine compounds (Cr), lipids, lactate, myoinositol, glutamate, and glutamine. Very recently, *IDH* mutations have also been discovered in gliomas,^18–20^ resulting in the MRS-detectable oncometabolite 2-hydroxygluterate (2HG).^18,19^

Increased levels of Cho have been detected in malignant tumors and are ascribed to an increased cellular membrane turnover.^21^ Decreased levels of NAA have also been detected in malignant tumors and are ascribed to the breaking of neuronal integrity.^21^ Every tissue, both normal and pathological, can be classified based on its MRS spectra (*i.e.* “biochemical fingerprint”).^22^ The “biochemical fingerprint” in normal tissue depends mainly on the location of the selected area and on the age of the patient. On the other hand, in the pathologic setting, the “biochemical fingerprint” varies depending on the type of tissue damage. In the presence of structural damage (trauma, tumor, degenerative diseases, gliosis etc.), the damaged tissue displays a specific pattern of metabolite concentration different from that obtained in the case of altered physiological conditions (*e.g.* interruption of blood flow) or biochemical or genetic problems.^22^ In the presence of a brain tumor, MRSI is able to identify the pathologic tissue with high accuracy where Cho, Cr and NAA are the main altered metabolites (an increase in the Cho levels, stability or decrease in Cr and decrease in NAA levels are usually seen).^16,22–24^ The Cho:NAA ratio and the Cho:Cr ratio are the most useful indices for the quantification of the metabolic abnormalities in tumor.^16,25^ Cho:NAA ratio >2 indicates metabolic abnormality in malignant tumors.^6,25^ A lactate peak and an increase in the lactate/lipid resonance with a reduction of all other metabolites can be detected in the treatment-induced necrosis, or in the necrotic portion of a tumor. Indeed, the concentration of lactate is correlated with anaerobic metabolism in hypoxic regions.^16^

## MRSI acquisition

MRSI acquisition is standardized for brain applications where two common pulse sequences are used: Point Resolved Spectroscopy (PRESS) and STimulated Echo Acquisition Mode (STEAM). Several studies have explored the use of MRSI at both 1.5 and 3 T. 3 T MRSI is becoming more readily available in recent years and has the advantage of nearly double the signal-to-noise ratio (SNR) and spacing between metabolite peak locations compared with 1.5 T MRSI. MRSI is performed following pre-contrast MRI acquisition but before gadolinium contrast administration. The volume of interest (VOI) encompasses the lesion (contrast enhancement on *T*_1_W, or hyperintense area on *T*_2_W,/FLAIR images) and the surrounding normal-appearing brain tissue. Outer volume saturation bands may be applied to avoid signal contamination from subcutaneous lipid, bone, and varying magnetic susceptibility effects that may compromise the quality of the spectra.^26–28^

Both PRESS and STEAM sequences generate localized spectra from a single VOI or voxel by applying three RF pulses and slice selective gradients. STEAM generates a signal from a rectangular or cubic voxel by using three orthogonal slice-selective 90 degree pulses. Similarly, PRESS generates a voxel by acquiring one orthogonal slice-selective 90 degree pulse followed by two 180 degree refocusing pulses. Voxel spectra generated by PRESS doubles the SNR but the voxel definition is not as precise as STEAM due to differences in the 90- and 180-slice selective pulses. The voxel size and location of the VOI can be easily controlled by adjusting slice selective pulses. Both techniques use a water signal suppression, since the peak from water is magnitudes greater than the peaks of interest, and often fat saturation can be applied in the region of interest. To suppress the water signal, CHEmically Selective Saturation is usually applied as a pre-scan technique.^26–28^

Two different spectroscopy approaches have been used in different fields of cancer successfully: single-voxel spectroscopy (SVS) and multivoxel spectroscopy. SVS samples a cubic or rectangular voxel (representing either the entire lesion or just its center) as the region of interest. SVS provides a global assessment of the metabolic content within the selected volume of tissue in short acquisition times.^29^ Thus, SVS does not provide information on spatial heterogeneity and does not help in the definition of the spatial extent of the lesion, causing it to be less useful for treatment planning (for RT or surgery) and follow-up assessment.^30^

Multivoxel MRSI, also known as chemical shift imaging (CSI), enables the simultaneous acquisition of multiple voxels in single or multiple slices. A large VOI is divided into smaller voxels, and CSI generates the spectrum of all voxels simultaneously. Hence, CSI allows for the acquisition of a matrix of multiple spectra, enabling *in vivo* 'mapping' of spatial variations of metabolites contained in both pathological and normal brain tissue.^29^ Thus, despite having a longer acquisition time than SVS, CSI is advantageous as it allows the metabolic examination of the entire tumor and surrounding tissues, and regions with the highest metabolic activity can be detected.^31^

## MRSI analysis

MRSI spectra can be analyzed both qualitatively and quantitatively to distinguish between tissue conditions, *i.e.* normal, benign, malignant, necrotic or hypoxic.

Multiple general and specialized software including vendor-based software are available to process both single-voxel and multivoxel MRSI data. These software (*i.e.* FuncTool Performance^30^ by General Electric and Syngovia by Siemens) can be used to process MRSI data and calculate area under the major peaks NAA, Cho, and Cr to generate color maps. Overlay images can be generated by applying the MRSI grid onto the corresponding CE-*T*_1_W or *T*_2_W/FLAIR anatomical images. Peak area ratios Cho/NAA and Cho/Cr can be calculated to generate color maps. All the currently available MRSI software can quantify metabolites that are well separated (*i.e.* no overlap in chemical shift dimension). When studying metabolites with complicated spin systems such as glutamate, glutamine, and 2HG, specialized software is required for the absolute quantification of these metabolites. Specialized software (including JMRUI,^32^ SIVIC,^33^ and LCModel^34^) may be used to quantify metabolite concentrations using a full Hamiltonian basis set or model spectra. When using the full Hamiltonian basis set to model input data within LCModel, the software generates a linear combination of the best metabolites to fit any given patient spectra, resulting in the quantification of raw concentrations of these metabolites. LCModel provides concentrations and Cramer–Rao lower bounds (concentrations +/− CRLB) estimate of variance for each metabolite in the basis spectrum. When assessing brain cancer, the first crucial step is to generate the model basis sets for all brain metabolites corresponding to the experimental scan conditions and prepare the MRSI data by coil combination, eddy current correction, and frequency alignment to avoid any possible frequency drifts; thereafter, LCModel can be used to estimate individual metabolite concentrations to generate color maps. Additional water unsuppressed MRSI signal and relaxation time constants will be helpful for generating color maps of absolute metabolite concentration.

## Clinical applications

Accurate clinical diagnosis, tumor delineation and treatment assessment are crucial to ensure optimal management of patients affected by brain tumors.

### MRSI in the clinical setting

Proton MRSI can add valuable information to conventional MRI in the clinical setting with regards to brain tumors and several disorders of CNS systems.^17^ Adding MRSI data to conventional MRI help in characterizing malignant lesions from normal tissue, differentiating high-grade gliomas from low-grade gliomas,^35^ and better defining non-enhancing lesions including diffuse intrinsic pontine gliomas (DIPG) which has the worst prognosis in pediatric patients.^36^ In a report by Hellstrom et al,^37^ authors re-evaluated MRI and MRSI (single voxel or multivoxel) in 208 patients with 70 non-neoplastic lesions, 43 low grade tumors, and 95 high grade tumors. In their study, they observed that MRSI yielded beneficial additional information in selected cases and may help in the evaluation of disease extent or location of hot spots.

### MRSI for identifying *IDH* gene mutations in gliomas

**Figure 1.**
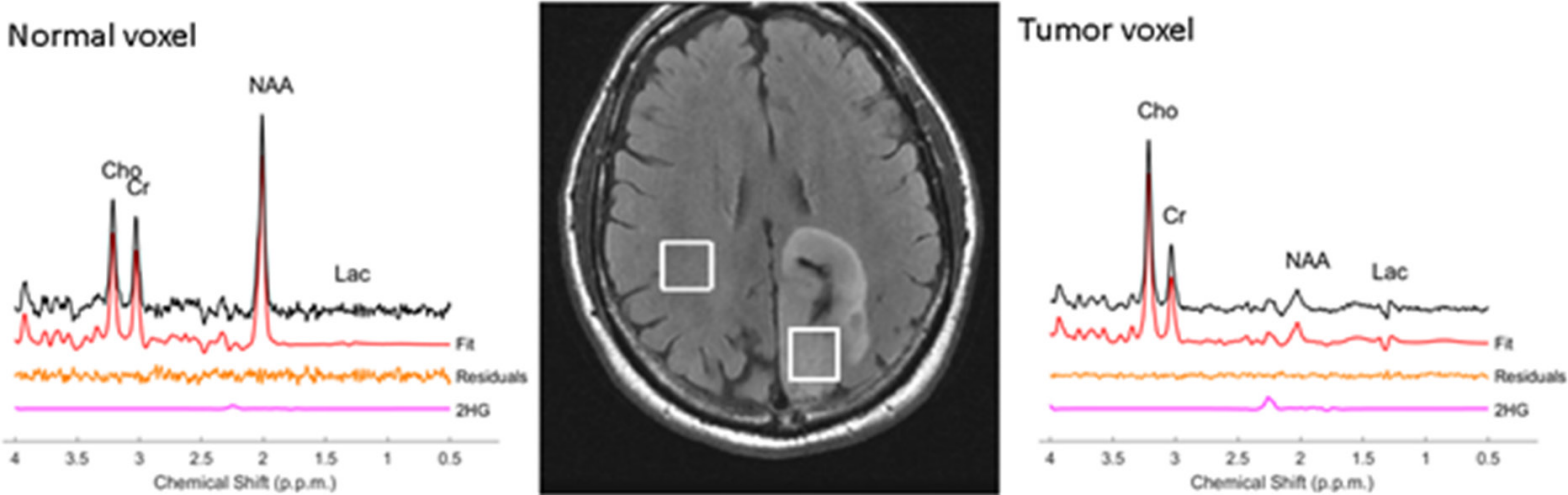
MRS for 2HG diagnostic status. *In vivo* 3 T single-voxel–localized PRESS spectra obtained in the brain patient with astrocytoma pathology and mutant IDH status: normal tissue (left) and tumor tissue (right) voxels MR spectra (experimental spectra, LCM Fit, residuals, and 2HG peak fit) along with the MRI showing voxel locations. Resonance at 2.25 ppm. indicates 2HG. The estimated 2HG metabolite concentration [2HG] is 8.1 mM (CRLB = 12%) in tumor tissue. 2HG, 2-hydroxygluterate; NAA, NAA, N-acetyl aspartate; IDH, isocitrate dehydrogenase; PRESS, PRESS, Point ResolvedSpectroscopy; CRLB, Cramer-Rao Lower Bound;

**Figure 2.**
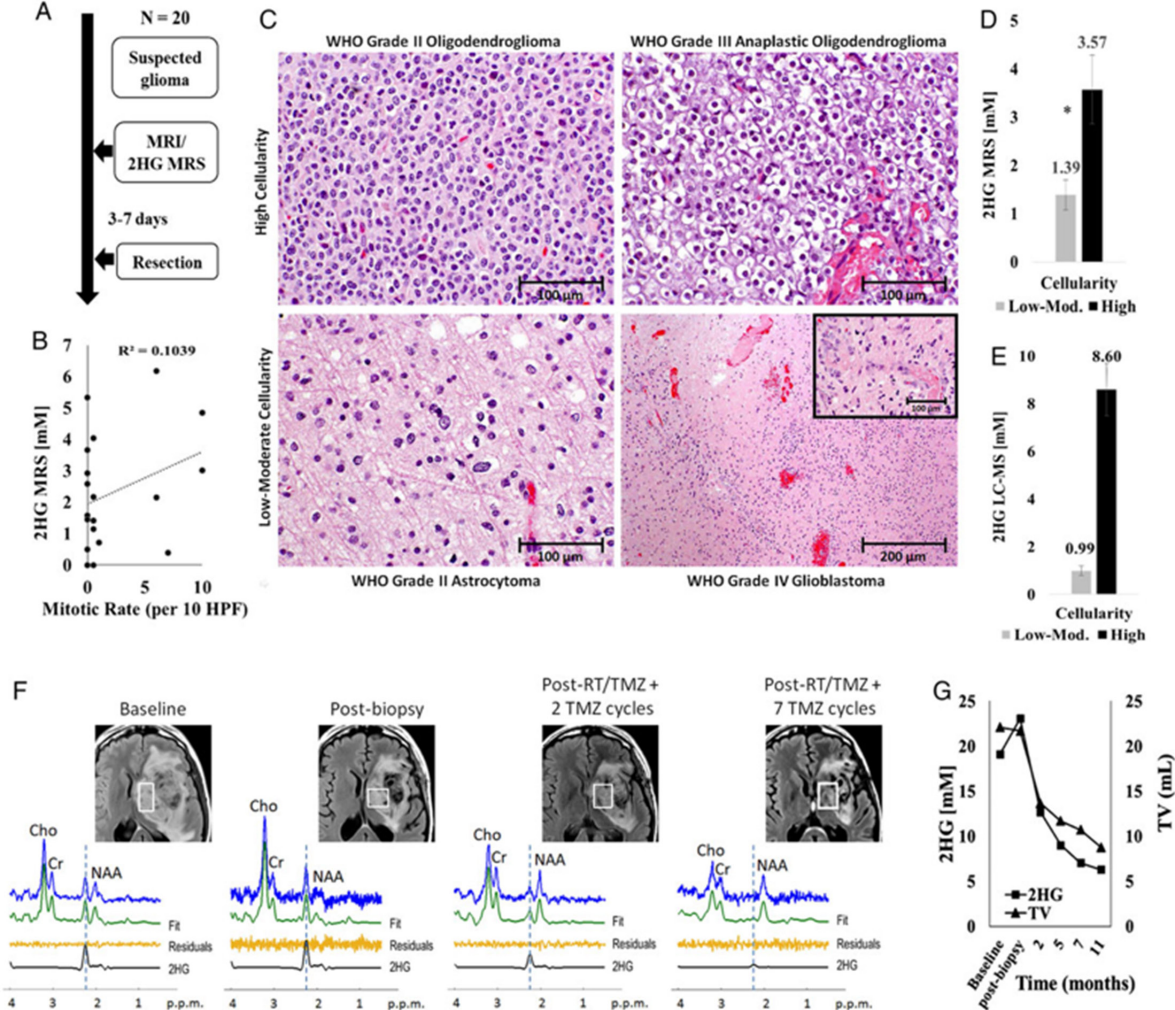
2HG MRS treatment monitoring. Correlation between conventional MR images and MR single voxel spectra from a patient with IDH2-mutant anaplastic astrocytoma prior to treatment (first panel), after a diagnostic tumor biopsy (second panel), and following radiation and chemotherapy (intensity-modulated radiation therapy with concurrent temozolomide followed by 12 monthly cycles of adjuvant temozolomide - third and fourth panels). The main 2HG peak is indicated at 2.25 of the x-axes and shows how 2HG concentration within the lesion progressively reduces during treatment, paralleling the reduction in tumor volume. (Reprinted from: De La Fuente MI, Young RJ, Rubel J, Rosenblum M, Tisnado J, Briggs S, ... Thakur, SB. Integration of 2-hydroxyglutarate-proton magnetic resonance spectroscopy into clinical practice for disease monitoring in isocitrate dehydrogenase-mutant glioma. Neuro Oncol. 2016. 18(2):283–290.) 2HG, 2-hydroxygluterate; IDH, isocitrate dehydrogenase

Nearly 80% of World Health Organization Grades II and III gliomas show the presence of mutation in the *IDH1* and *IDH2* genes that regulate the omonimous enzymes. These enzymes are important for converting isocitrate to form α-ketoglutarate. Oncologic patients with *IDH1* and *IDH2* mutant enzymes have a gain-of-function to further convert the α-ketoglutarate to 2HG, an oncometabolite which has been described as a useful oncomarker in the diagnosis and follow-up in glioma patients.^38^ The presence of 2HG can help to differentiate gliomas from non-neoplastic processes (*e.g.* demyelinating disease). As patients with *IDH1* mutation have a greater 5 year survival rate than patients with wild-type *IDH1* gliomas (93% *vs* 51%) when correcting for age,^31^ 2HG is also an important prognostic marker.^18^ As opposed to what happens in patients affected by hydroxyglutaric aciduria metabolic disorders, in patients with *IDH*-mutant gliomas, 2HG levels increase in tumor tissue but not in blood levels. This is why the ability to detect 2HG makes MRSI a very important diagnostic tool.^18,31^ In previous studies, the accumulation of 2HG in brain gliomas as detected on MRSI has been correlated with mutations in *IDH1*^18,31^ and *IDH2*. A representative example of a patient with mutant IDH tumor showing the positive 2HG peak is shown in Figure 1. 2HG is defined as a positive peak when there is a detectable spectral peak at 2.25 ppm at an estimated concentration ≥ 1mM with CRLB ≤ 30%^19^. 2HG is absent in the normal appearing tissue. Based on this criteria, 2HG peak is defined as negative in the normal appearing tissue MR spectra. Of note, de la Fuente et al^19^ showed that the sensitivity of MRSI for detecting the 2HG oncometabolite is highly dependent on tumor volume. In particular, MRSI is significant less sensitive for a tumor volume of 3.4 ml compared with a tumor volume of 8 ml (generally considered the lower limit of MRSI resolution), with the sensitivity ranging from 8% for tumor volumes <3.4 ml to 91% for larger tumors (>8 ml). This study showed lower 2HG detection sensitivity in larger tumors (>8 ml) due to the tumor cellularity within the selected volume. 2HG detection in small tumors with lower voxel volumes (<3.4 ml) may be improved by increasing the number of excitations, which increases the scanning time. In situations of longer scan times, patient motion may introduce errors, and hence it is not clinically feasible. Further improvements in hardware (32- or 48-channel coils) and software (*e.g.* advanced denoising for SNR improvement or artifact reduction)^39,40^ can help to decreas false negatives and improve clinical diagnosis. 2HG has also been investigated as a biomarker for monitoring tumor response to treatment where 2HG may be lowered upon successful therapy. Indeed, de la Fuente et al^19^ reported decreased intratumoral 2HG levels during cytoreductive therapy (Figure 2), suggesting that 2HG-MRSI may be a useful non-invasive imaging biomarker for monitoring cytoreduction in *IDH*-mutant solid tumors. This suggests that 2HG-MRSI may be an important diagnostic tool for patients with *IDH*-mutant gliomas receiving standard treatments or *IDH* inhibitors in clinical trials. The reduction in the 2HG metabolite concentrations closely parallels the reduction in the tumor volume as shown in Figure 2. 3D MRSI of 2HG was also shown to be valuable for radiotherapy planning.^41^ In 47% of patients, 2HG volume was larger than FLAIR volumes and hence appears to have an important implication in RT planning.

### MRSI delineation of target tumor volumes for RT assessment

**Figure 3.**
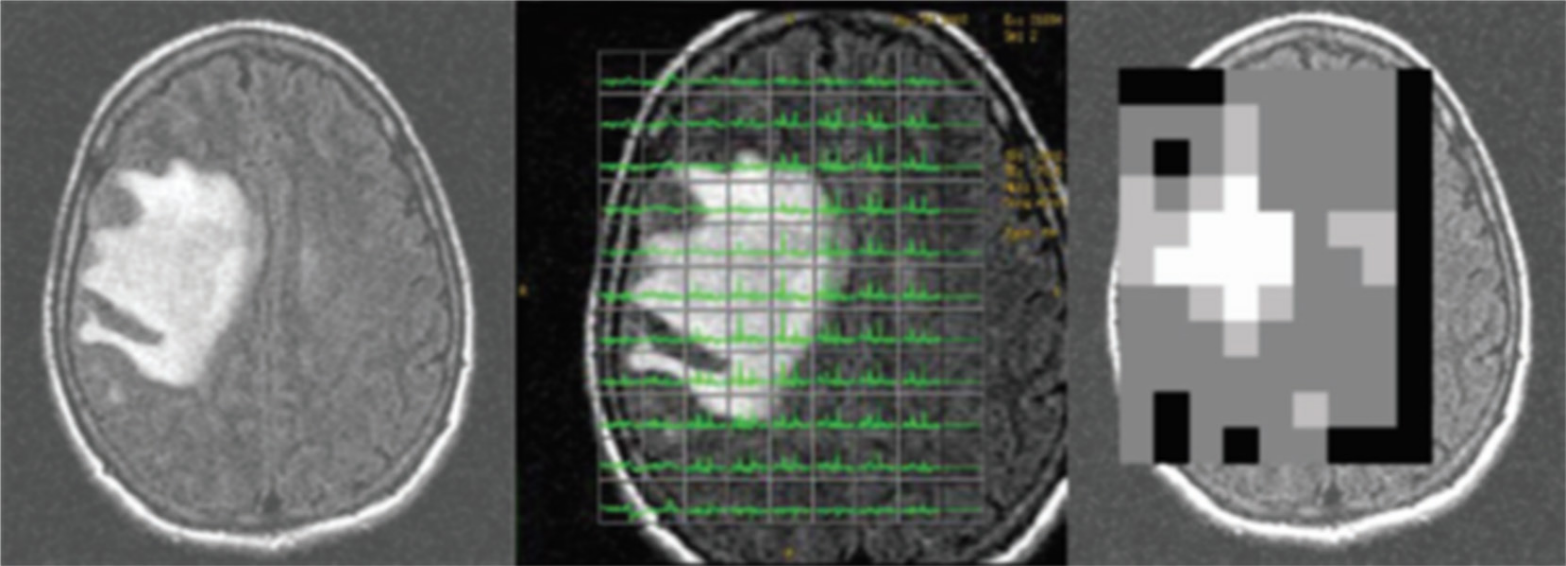
MRSI incorporation in treatment planning. (a) FLAIR image of a right frontal glioma. (b) Superimposition of multivoxel MRSI over the image. (c) Conversion of Cho/Cr ratio into a grayscale. Reprinted from: Narayana A, Chang J, Thakur S, Huang W, Karimi S, Hou B, Kowalski A, Perera G, Holodny A, Gutin PH. Use of MR spectroscopy and functional imaging in the treatment planning of gliomas. Br J Radiol. 2007;80(953): 347–354. FLAIR, fluid attenuatedinversion recovery; MRSI, magnetic resonance spectroscopy imaging.

**Figure 4.**
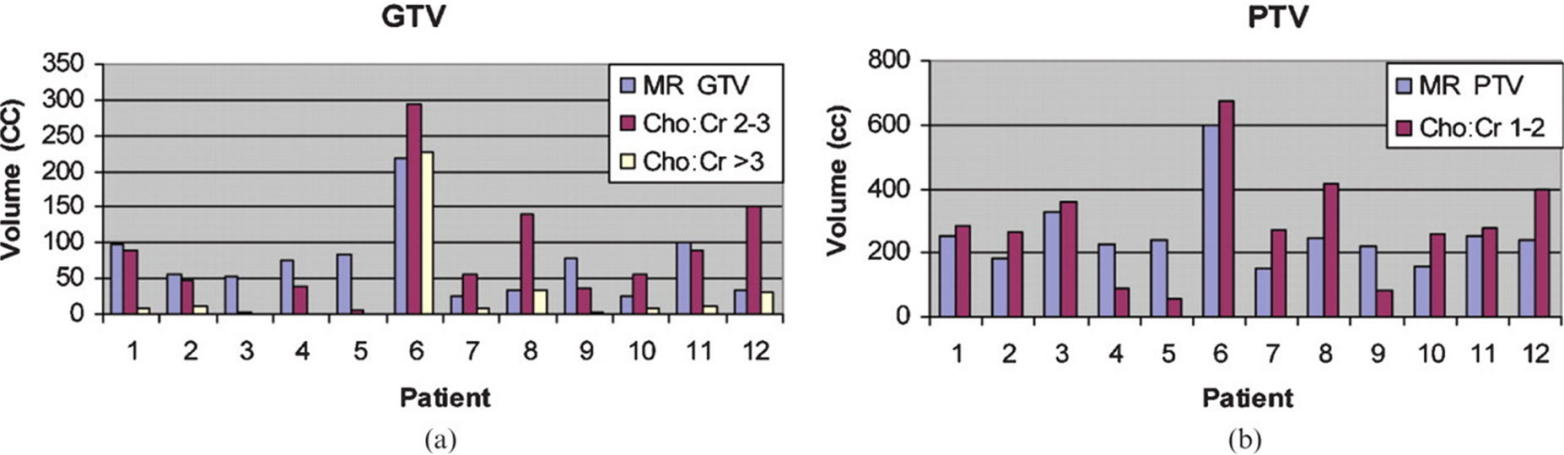
Comparison of PTVs with MRI and MRSI defined volumes. Reprinted from: Narayana A, Chang J, Thakur S, Huang W, Karimi S, Hou B, Kowalski A, Perera G, Holodny A, Gutin PH. Use of MR spectroscopy and functional imaging in the treatment planning of gliomas. Br J Radiol. 2007;80(953):347–354. GTV, gross tumor volume;MRSI, magnetic resonance spectroscopy imaging; PTV, plannedtarget volume.

High-grade lesions often have a heterogeneous composition, increasing the risk that a non-specific or lower-grade region of the tissue is sampled during biopsy.^15^ MRSI can be used to identify the area that would be the best target for biopsy (*i.e.* the area of highest metabolic activity).^42–47^ Elevated Cho levels and low NAA levels in an area of the lesion indicate the presence of active tumor, whereas areas with low Cho and NAA levels may be associated with less metabolic activity (*i.e.* lower grade), necrosis, astrogliosis, macrophage infiltration, or mixed tissue.^15^ In Narayana et al, different peak resonances or MRSI metabolites were utilized for preparing the fusion images of MRSI onto MRI for RT planning (Figure 3). The authors were able to successfully define the target volume in all 12 cases based on the Cho/Cr ratio. There was no correlation between MRI-defined planning target volumes and MRSI-defined Cho/Cr > 2 volumes. However, MRSI-defined gross tumor volumes (Cho/Cr > 3) were smaller by 40% compared with post-contrast *T*_1_W imaging-defined gross tumor volumes (Figure 4).

Localized RT represents the best non-surgical therapeutic approach in brain tumors. The aim in localized RT is to maximize the therapeutic dose to the regions of highest metabolic and cellular activity (gross total volume) while sparing/administering minimal dose to perilesional tissue that might include tumor infiltration (clinical target volumes) or normal brain tissue.^48–50^ However, the delineation of tumor boundaries represents one of the main challenges in the management of brain tumors due to their infiltrative nature. Conventional CE-*T*_1_W and *T*_2_W/FLAIR MR sequences are not sensitive in the definition of the real extent of these lesions, which are characterized by a diffuse growth pattern.^51^ Further, *T*_1_W contrast enhancement and/or *T*_2_W hyperintensity are not specific indicators since they are present also in necrosis, edema or treatment-related changes. In this setting, MRSI can add important information to MRI about the areas of tumor activity in RT treatment planning.^50^ Many authors have described the ability of MRSI to detect areas of tumor infiltration beyond contrast enhancement or within edema.^52–55^ In those areas, a high CNR ratio is suggestive of a highly proliferative, non-neuronal cell population as it comprises markers describing membrane lipid biosynthesis (Cho) and neuronal integrity (NAA). Some authors have also shown that MRSI facilitates a better definition of the target volume, since the area of metabolic alteration can exceed the region of altered signal on anatomical *T*_2_W images.^49,56^ Pirzkall et al found that metabolically active tumor provided by MRSI extended outside the region at risk, as estimated on T2 in 88% of patients by as many as 28 mm.^57^ The metabolic information from MRSI can be added to the information from conventional anatomical MRI, leading to a higher coverage of recurrent contrast-enhancing tumor and a more precise estimation of disease extent.^58^ Cordova et al showed that MRSI-based target volume identified significantly different regions of microscopic tumor infiltration than conventional clinical target volumes and exhibited better coverage of contrast-enhancing tumor at recurrence in two out of seven patients.^11^ In a multisite pilot study which aimed at establishing feasibility and safety of dose-escalated RT based on metabolic abnormalities in patients with GBM, Gurbani et al developed the Brain Imaging Collaboration Suite which is a cloud platform that combines whole-brain MRSI and MRI data and allows members from different institutions to define RT targets. In their series, they did not detect any severe toxicity in 18 patients treated using targets created in Brain Imaging Collaboration Suite.^59^ However, there is little clinical evidence in favor of an irradiation based on a bigger target volume defined on metabolic features.

### MRSI for evaluation of RT

Radiation necrosis represents a localized reaction to RT in 3–24% of glioma patients treated with adjuvant RT.^60^ On conventional CE-*T*_1_W and *T*_2_W/FLAIR images, it is difficult to differentiate radiation necrosis from tumor recurrence/progression as both show a T2 hyperintense signal and T1 enhancement after gadolinium administration. Several authors^54,61–64^ have shown that MRSI can help in the discrimination of RT-induced changes and tumor recurrence. Specifically, increased Cho levels (Cho levels relative to Cho signal in normal brain regions, Cho/Cr or Cho/NAA ratios) are indicative of the presence of tumor tissue/recurrence while reduced Cho (and Cr) levels suggest that the anatomical signal changes observed are related to radiation necrosis. MRSI has also shown to be useful to differentiate radiation-induced changes from tumor recurrence after γ knife radiosurgery and brachytherapy.^65^

**Figure 5.**
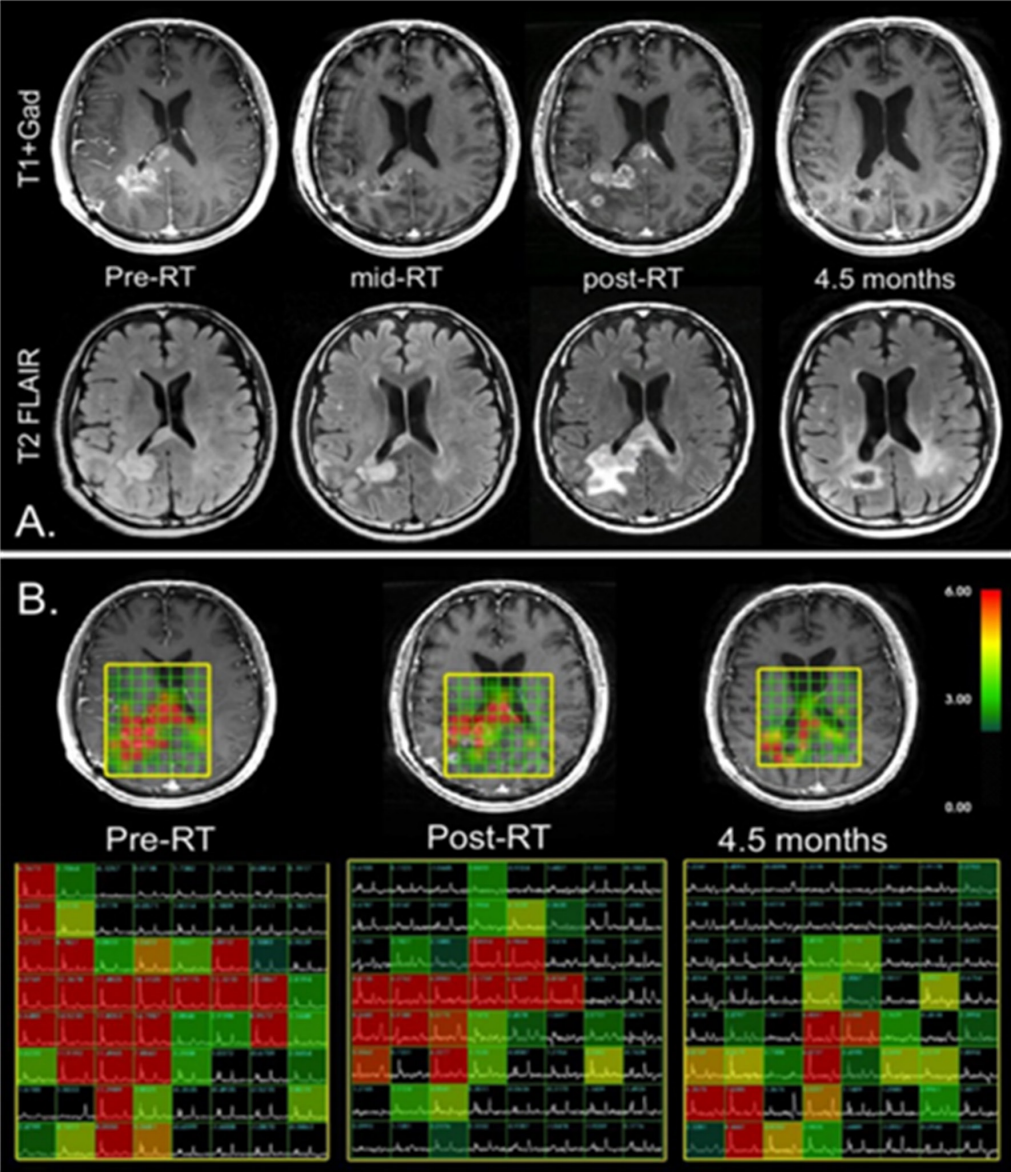
MRSI for evaluation of radiation therapy. Correlation between *T*_2_ weighted FLAIR images (A), *T*_1_ weighted post-gadolinium images (B - top) and metabolic MRSI imaging data (B-bottom) from four serial examinations of a patient affected by GBM treated with RT, temozolomide and enzastaurin. The early post-RT exam showed an increase in contrast enhancement as well as T2 abnormal signal of the lesion, but these decreased over time, suggesting pseudoprogression. In regard to the MRSI data, the region of abnormal CNI decreased with time and intensity during the examinations, indicating response to therapy. (Reprinted from: Lupo JM, Nelson SJ. Advanced magnetic resonance imaging methods for planning and monitoring radiation therapy in patients with high-grade glioma. Semin Radiat Oncol. 2014;24(4):248–258.). CNI, Cho to NAA index; FLAIR,fluid attenuated inversion recovery; GBM, glioblastoma multiforme; MRSI, magneticresonance spectroscopy imaging, RT, radiation therapy.

The changes in tumor volume are usually evaluated using conventional MRI to assess the stability, remission or progression of the disease after therapy.^64^ In this setting, MRSI can anticipate metabolic tumoral changes before dimensional changes, whereby decreased Cho levels after RT are suggestive of partial remission while stable or increased Cho levels are suggestive of disease progression (Figure 5).^12,50,66,67^ An interesting study showed that MRSI at baseline MRI can predict the onset of new contrast enhancement in post-treatment MRI in patients with GBM.^68^

Laprie et al^69^ examined the association between MRSI metabolite ratio “CNR“ abnormalities and the sites of relapse from patients affected by GBM treated with Tipifarnib and RT in patients enrolled in a prospective clinical Phase I trial. In their series, they showed that metabolically active regions at MRSI were predictive for the site of post-RT relapse, suggesting that the correlation between MRSI and MRI data in the definition of RT target volumes could increase GBM local control.

As lactate accumulation might be considered as a surrogate marker of hypoxia, Deviers et al^70^ evaluated whether the pre-RT distribution of lactate-to-N-acetyl-aspartate ratio (LNR) in GBM could predict tumor response to RT. They observed contrast enhancement at relapse in tumoral regions with LNR > 0.4, thus suggesting that higher LNR were associated with the radioresistant parts of the tumor.

A promising strategy to improve post-RT local control of GBM is represented by the “dose-painting approach,” which involves the heterogeneous irradiation of tumor targets, with higher doses administrated to the potential areas of radioresistance.^71,72^ Those areas are delineated by combining metabolic and conventional MRI data.

In particular, Ken et al^73^ in their multi-institutional Phase-III clinical trial proposed a new approach to point out potential radioresistant areas for dose painting to guide simultaneous integrated boost in intensity-modulated radiation therapy. In their series, they identified target volumes integrating metabolic maps to conventional MRI data in order to perform dose painting by contours for higher irradiation of regions of high-risk of relapse while optimizing the irradiation of organs at risk, thus reaching a compromise between radiation necrosis, neurocognitive impairment, and tumor control.

Also, Laprie et al,^74^ in the SPECTRO-GLIO trial, which is a dose-painting trial, hypothesized that dose escalation on metabolic abnormal tumoral regions (*i.e.* Cho/NAA ratio >2) detected with MRSI improved local control without increasing the dose to organs at risk.

Furthermore, MRSI has been shown to be useful in evaluating and monitoring radiation-induced injuries in normal brain tissue. Decreased NAA and Cho levels have been detected in non-tumoral regions 1–4 months after RT (20–50 Gy).^75,76^ Recently, there was a report published by Cordova et al, in efforts toward incorporating MRSI as a metric for radiation therapy planning in patients with glioblastomas.^11^ With recent developments in hardware and software,^11^ typical resolutions of 4.4 × 4.4 × 5.6 mm within acquisition times of less than 20 min were possible for treatment planning. A few studies have proposed real-time volumetric echoplanar imaging navigators to manage patient motion and correct it while using echo planar-based acquisition schemes. A recent study by Moser et al^77^ assessed intra-/intersubject coefficients of variation and intraclass correlations over the whole 3D volume using 3D-FID-MRSI navigators for real time motion and shim corrections.

## Limitations and novel applications

MRSI has a few limitations. Firstly, it requires a relatively long acquisition time (>10–15 min) for high spatial resolution metabolic imaging and tissue metabolites detection with higher sensitivity in brain tumors. Because of that, numerous fast acquisition methods such as echoplanar and reconstruction strategies from undersampled data have been developed in order to shorten the scan time and increase the adoption of MRSI into routine clinical protocols.^78^ Additionally, factors like poor shimming and lipid contamination due to skull-based fat must be controlled to avoid a low-quality spectrum. Although MRS can be used to detect multiple metabolites, it requires some optimization for the detection of metabolites other than conventional or dominantly used metabolites such as NAA, Cho, Cr, and lactate. In their meta-analysis, Wang et al^79^ reported that the overall sensitivity and specificity of MRS are 80.05% (95% CI: 75.97, 83.59) and 78.46% (95% CI: 73.40, 82.78), respectively, for the evaluation of brain tumor patients. With available high-performance field gradients and multichannel receiver coils, further work using echoplanar spectroscopic imaging in combination with advanced reconstruction methods can be employed to achieve high spatial resolution MRSI data within clinically feasible time. Additionally, when MRSI coverage area is large, then modern radiofrequency coils with shim elements can also facilitate excellent MRSI line shape quality, resulting in accurate quantification of brain metabolites to assess radiation treatment.

**Figure 6.**
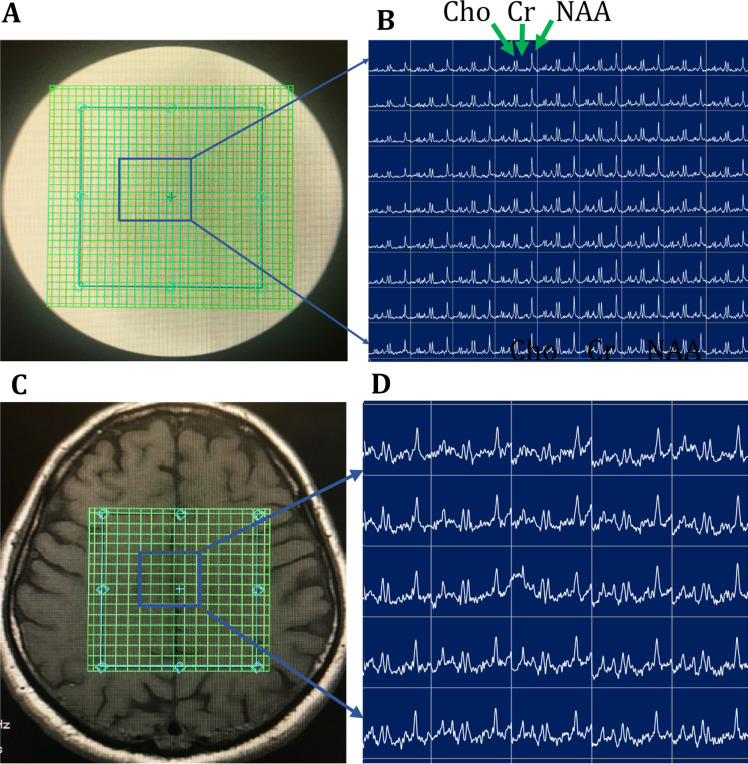
T1 MR images overlaid with the MRSI grid and corresponding semi-LASER spectroscopic imaging data using symmetric echo planar spatial encoding obtained within a scan time of 1-2 minutes. (A, B) GE Braino MRS phantom (C, D) Volunteer. Acquisition parameters: Repetition time, 2000 ms; echo time, 35 ms; matrix size, 32 x 32 for phantom; 16 × 16 for *in vivo*; field of view, 80 mm; number of averages, two for phantom and four for volunteer; slice thickness = 15 mm. Note that uniform excitation of major metabolites was found to be possible within a rapid scan time^83^ . MRSI, magnetic resonance spectroscopy imaging.

The reproducibility of MRSI in the clinical setting depends on a couple of factors, including hardware, patient immobilization, baseline irregularities, and accuracy of volume position. Coefficients of variation, 100% times the standard deviation divided by the mean, is usually used to evaluate the reproducibility of metabolite peak levels and ratios.^26^ The reliability of MRSI depends on SNR, spatial resolution, and sharper linewidths. Higher field strength MR systems (*i.e.* 7 and 9.4 T) can provide more reliable quantification of metabolites with overlapping peaks; however, their efficacy still needs to be proven.^80,81^ Emerging techniques, such as hyperpolarized Carbon-13 MRSI, can also provide additional information in the study of brain tumor metabolism.^82^ It may be feasible to integrate measurements of steady-state metabolism by 2HG-MRS with an investigation of metabolic-flux using 13C- HP-MRS. With the discovery of new iPRES coils and semi-LASER pulse sequences, it may be possible to acquire 2D or 3D MRSI with uniform B0 and B1 homogeneity over the MRSI volume. Additionally, one can apply echoplanar spectroscopic imaging techniques (Figure 6) for speeding up 2D/3D acquisition times using semiLASER volume localization, making this tool clinically feasible. Among several reports published, recently, a report by Steel et al^84^ demonstrated the potential of a new k-space trajectory in volunteers using metabolite-cycled density-weighted concentric rings in combination with volume localization with semiLASER to enable 2D high spatial resolution MRSI.

## Deep learning MRSI

The concept of deep learning networks has been extended to spectroscopy to obtain super-resolution MRSI. Deep learning techniques might provide super resolution MRSI images similar to MRI and thus may help tailor treatment planning. In a recent study by Iqbal et al,^85^ spatial resolution in MRSI was further improved using novel deep learning methods to reconstruct high resolution MRSI images using information from anatomical *T*_1_W MR images. Low resolution MRSI and T1 images were used as input to this model and generated super resolution MRSI data as output. In this study, the authors reported a novel densely connected UNet (D-UNet) architecture capable of producing super-resolution MRSI images which are comparable in quality and quantitatively to simulated and *in vivo* high resolution MRSI. The authors concluded that deep learning methods can generate high quality super resolution data using low-resolution data obtained both at 3 and 7 T field strengths. Further work is needed on perspective *in vivo* data, and *in vitro* and *in vivo* validation.

## Conclusions

MRSI is a promising non-invasive technique that provides both tumor metabolic information and insight into the physiology of malignant transformation in brain tumors. Its clinical applications have been heavily investigated for cancer diagnosis and monitoring treatment response, and quite recently for non-invasive detection of mutated *IDH* gene status in gliomas. MRSI is also a valuable tool to improve tumor delineation for radiation therapy planning; however, its impact on clinical practice needs further evaluation.
